# A Simple Sketch Symbolizing Self-Reliance

**DOI:** 10.3201/eid2211.AC2211

**Published:** 2016-11

**Authors:** Byron Breedlove

**Affiliations:** Centers for Disease Control and Prevention, Atlanta, Georgia, USA

**Keywords:** art science connection, emerging infectious diseases, art and medicine, about the cover, infectious diseases, A Simple Sketch Symbolizing Self-Reliance, Walden, or, Life in the Woods, Walden Pond, Henry David Thoreau, Sophia Thoreau, respiratory infections, tuberculosis, public health

**Figure:**
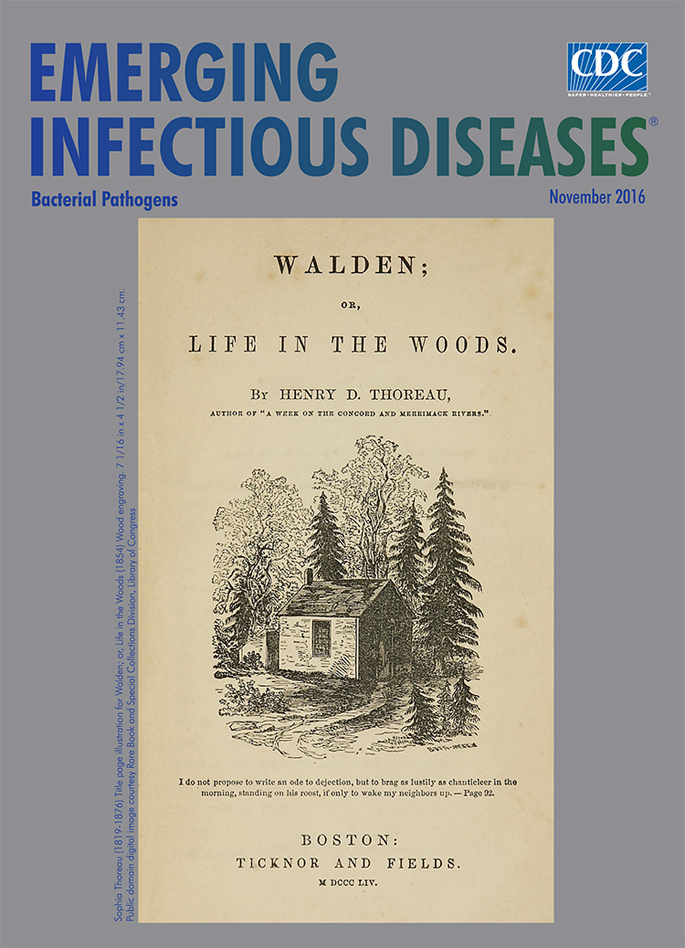
**Sophia Thoreau (1819–1876) Title page illustration for *Walden; or, Life in the Woods* (1854) Wood engraving, 7 1/16 in × 4 1/2 in/17.94 cm × 11.43 cm.** Public domain digital image courtesy Rare Book and Special Collections Division, Library of Congress.

Little cabin in the woods

Little man by the window stood

Saw a rabbit come hoppin’ by

Knockin at his door…

Henry David Thoreau (1817–1862) is remembered and celebrated as an essayist, poet, philosopher, and naturalist; he was also a surveyor, historian, antislavery activist, tax resister, and teacher. Thoreau’s extensive writings are collected in more than 20 volumes, including books, articles, essays, journals, and poems. In his best known book, *Walden; or, Life in the Woods*, published in 1854, Thoreau detailed his day-to-day experiences during the 2 years, 2 months, and 2 days he dwelled in a small, rustic cabin that he constructed near Walden Pond. This 64-acre lake is near the town of Concord, Massachusetts, USA, close to where Thoreau was born and lived most of his life.

Initially a modest success, the book sold 2,000 copies within 5 years but then went out of print until 1862, the year Thoreau died. Today it is among the best known works of American literature. Its chapters consist of the author’s ruminations on human existence, society, government, economics, nature, and other topics that were inspired by his daily observations on local events or his close-up encounters with nature. In an essay about *Walden*, “A Sage for All Seasons,” American writer and critic John Updike opined, “A century and a half after its publication, *Walden* has become such a totem of the back-to-nature, preservationist, anti-business, civil-disobedience mindset, and Thoreau so vivid a protester, so perfect a crank and hermit saint, that the book risks being as revered and unread as the Bible.”

Thoreau died of tuberculosis in May 1862, a disease that first manifested itself in 1835 while he was a student at Harvard College. He had endured several years of declining health, starting with a bout of bronchitis. Many of his large projects were left behind in various stages of completion and would have been lost to history were it not for the industrious work of his younger sister, Sophia Thoreau.

In *Walden*, Henry Thoreau wrote’ “For more than five years I maintained myself thus solely by the labor of my hands, and I found that, by working about six weeks in a year, I could meet all the expenses of living.” Sophia, in contrast, managed the family’s business after their father’s death, and according to the Concord Museum “helped manage her brother’s literary legacy in the years immediately following his death, and she is largely responsible for the preservation of his material legacy as well.” She, too, is remembered as having been a teacher, naturalist, and antislavery activist. Sophia also gardened and cultivated flowers, and she served as caretaker for her brother as he succumbed to tuberculosis.

Sophia Thoreau’s reputation as an artist, which is not without critics who considered her work simplistic, endures in large measure because it was her drawing of the cabin by the pond that Henry selected for the cover page of *Walden; or, Life in the Woods.* According to an article published by the Thoreau Society, this simple sketch “has become a symbol of individuality and self-reliance throughout the world.” In her sketch, Sophia depicted the cabin nestled among the thrusting evergreens and lacy deciduous trees. A path from the door shows the starting—and ending—point for Henry’s long rambles in the countryside or nearly daily walks to Concord. This path would also take visitors to the rustic cabin where Henry enjoyed his largely solitary life: “I had three chairs in my house; one for solitude, two for friendship, three for society.”

Perhaps Henry Thoreau’s active lifestyle and love of the outdoors helped him live with tuberculosis—the leading cause of death during his lifetime—for decades. This illness also claimed his grandfather, his father, and his older sister. His brother John, who died from tetanus, was also living with tuberculosis. That so many members of the Thoreau family died of tuberculosis is not remarkable: in the 1800s, living conditions in the United States contributed to outbreaks of infectious diseases such as tuberculosis, dysentery, cholera, malaria, pneumonia, typhoid fever, and whooping cough.

Since the initial publication of *Walden; or, Life in the Woods*, ensuing advances in sanitation, the advent of immunizations, the development of antibiotics, and improvements in care and diagnosis have greatly reduced deaths from diseases caused by many of the respiratory pathogens that were so devastating during the 1800s. However, during the 21st century, myriad outbreaks of novel respiratory tract infections that could potentially cause epidemics or pandemics, including Middle East respiratory syndrome, severe acute respiratory syndrome, H1N1 influenza, and invasive pneumococcal disease, underscore the value of continuously advancing and assessing public surveillance, diagnosis, prevention, and treatment strategies and responses.

## References

[1] Fauci AS, Morens DM. The perpetual challenge of infectious diseases. N Engl J Med. 2012;366:454–61. 10.1056/NEJMra1108296 22296079

[2] Harding W. Thoreau and tuberculosis. Thoreau Society Bulletin. Bulletin 186, Winter 1889 [cited 2016 Aug 29]. https://archive.org/stream/thoreausociety1989186unse/thoreausociety1989186unse_djvu.txt

[3] Herrick GL. Sophia Thoreau–“Cara Sophia” The Concord Saunterer. 1978: Vol. 13, No. 3 (Fall 1978). p. 5–12. The Thoreau Society, Inc. [cited 2016 Aug 29]. https://www.jstor.org/stable/23393396?seq=1#page_scan_tab_contents

[4] Manoli-Skocay C. A gentle death: tuberculosis in 19th century Concord [cited 2016 Aug 31]. http://www.concordlibrary.org/scollect/essays/death.html

[5] Thoreau HD. The Library of America edition: A week on the Concord and Merrimack Rivers; Walden, or, life in the woods; the Maine woods; Cape Cod [cited 2016 Aug 29]. http://www.eldritchpress.org/walden5.pdf

[6] Centers for Disease Control and Prevention (CDC). Control of infectious diseases. MMWR Morb Mortal Wkly Rep. 1999;48:621–9. 10458535

[7] Updike J. A sage for all seasons. The Guardian, June 25, 2004 [cited 2016 Aug 26]. https://www.theguardian.com/books/2004/jun/26/classics

[8] Wayne TK. Encyclopedia of transcendentalism. New York: Facts on File; 2014. p. 281–2.

[9] Zumla A, Hui DS, Al-Tawfiq JA, Gautret P, McCloskey B, Memish ZA. Emerging respiratory tract infections. Lancet Infect Dis. 2014;14:910–1. 10.1016/S1473-3099(14)70899-0 25189348 PMC7128723

